# Guillain-Barré Syndrome: Natural history and prognostic factors: a retrospective review of 106 cases

**DOI:** 10.1186/1471-2377-13-95

**Published:** 2013-07-22

**Authors:** Inés González-Suárez, Irene Sanz-Gallego, Francisco Javier Rodríguez de Rivera, Javier Arpa

**Affiliations:** 1 Section of Neuromuscular diseases, Department of Neurology, La Paz University Hospital, Paseo de la Castellana, Madrid 261.28046, Spain

**Keywords:** Guillain-Barre, Natural history, Prognostic factors, Peak flow

## Abstract

**Background:**

Guillain-Barre syndrome (GBS) is characterized by acute onset and progressive course, and is usually associated with a good prognosis. However, there are forms of poor prognosis, needing ventilatory support and major deficits at discharge. With this study we try to identify the factors associated with a worse outcome.

**Methods:**

106 cases of GBS admitted in our hospital between years 2000–2010 were reviewed. Epidemiological, clinical, therapeutical and evolutionary data were collected.

**Results:**

At admission 45% had severe deficits, percentage which improves throughout the evolution of the illness, with full recovery or minor deficits in the 87% of patients at the first year review. Ages greater than 55 years, severity at admission (p < 0.001), injured cranial nerves (p = 0.008) and the needing of ventilator support (p = 0.003) were associated with greater sequels at the discharge and at the posterior reviews in the following months. 17% required mechanical ventilation (MV). Values < 250 L/min in the Peak Flow-test are associated with an increased likelihood of requiring MV (p < 0.001).

**Conclusions:**

Older age, severe deficits at onset, injured cranial nerves, requiring MV, and axonal lesion patterns in the NCS were demonstrated as poor prognostic factors. Peak Flow-test is a useful predictive factor of respiratory failure by its easy management.

## Background

The term Guillain-Barré syndrome (GBS) includes a set of clinical syndromes (GBS) with a common pathophysiological basis; an acute inflammatory polyneuropathy with an autoimmune etiology [1-3]. Although usually characterized by a progressive flaccid paralysis with areflexia a wide range of motor, sensory and autonomic symptoms could be seen [1-4]. In general, the diagnosis is based on clinical criteria [4-7]; nevertheless, the presence of suggestive findings in the complementary test as demyelinising changes in the nerve conduction studies (NCS) or albuminocytological dissociation in the cerebrospinal fluid (CSF), help to confirm the diagnosis [1].

The worldwide incidence of GBS is reported to be 0.6-2.4 cases per 100,000 per year [8-15]. The classic form, the acute inflammatory demyelinating polyradiculoneuropathy (AIDP), is the most frequent subtype in Europe, which accounts for 90% of GBS cases [2]. Other subtypes like the axonal forms or the Miller-Fisher syndrome (MFS) [16,17] are less common.

The prognosis is usually good, showing a complete functional recovery or with minimal deficits in the 90% of patients 1 year after the onset of illness [13,18]. Several factors have been identified as predictors of poor outcome [13,14,19-21]. Death rate is described to be between 1-18% [14,15]. This study aimed to describe the epidemiological, clinical, laboratory, and electrodiagnostic features, as well as to identify the predictive factors of worse prognosis in the GBS or its subtypes.

## Methods

A retrospective review of the medical records of patients admitted at La Paz University Hospital (Madrid, Spain) with the diagnosis of GBS between 2000–2010 was made. 106 fulfilled levels 1, 2 or 3 of diagnostic certainty for GBS/MFS described by Sejvar et al. [7]. All demographic, clinical, laboratory and electrophysiological data were recorded, as well as outcome and treatment.

Severity at admission was assessed by the Medical Research Council (MRC) sum score, valuing the strength from 0 to 5 in 4 muscles (proximal and distal) in both upper and lower limbs on both sides, so that the score ranged from 40 (normal) to 0 (quadriplegic) and by the GBS disability score advocated by Hughes et al. [22]. Cranial nerve involvement was considered separately by the affectation of oculomotor, facial and bulbar nerves. Respiratory weakness was assessed first by the value obtained at the peak expiratory flow meter (Peak Flow), as well as the need for mechanical ventilation throughout the evolution. Sensory disturbances, autonomic alteration or pain presence were also assessed.

Serological screening for preceding infections was recorded, including Herpes Simplex virus (HSV), Varicella-Zoster (VZV), cytomegalovirus (CMV), Epstein-Barr virus (EBV), *Mycoplasma Pneumoniae*, B and C hepatitis virus, *Haemophilus Influenzae* and, in selected cases, stool and *Campylobacter Jejuni* determination. The CSF was analyzed for cell count, glucose and protein concentration.

Neurophysiological studies were evaluated in accordance with the criteria of Hadden et al. [23,24]. As in a retrospective study, not in all cases were the same nerves measured. Also, electromyography studies (EMG) with concentric needle electrodes were made to evaluate the axonal loss (fibrillation, positive sharp wave…).

The evaluation of the functional impact was graded by the GBS disability score [22] during the discharge from the neurology or the rehabilitation department, and at the third, sixth and twelfth month in the outpatient clinic.

The study was approved by the Research Ethics Committee of La Paz Hospital, Madrid, Spain.

All statistical analyses were performed using the SPSS 12 for Windows program (Chicago, IL, E.U.A.). For univariate analysis, Chi-square test for dichotomic variables was used. For continuous variables, t-Student test in parametric variables or U Mann-Withney with the non-parametric ones were used.

## Results

There was no difference between genders (ratio male/female 1.07), with a mean onset age of 43.7 ± 23 years (range 0–85). Demographic and clinical data are summarized in Table 1. There was a seasonal rebound in winter, when 41% of patients were diagnosed. Most patients had an infectious antecedent preceding the onset of the weakness, being the most frequent respiratory tract infection (38%); at least 30% did not present a previous infectious disease [25]. The mean of days since the start of the infectious sickness until the polyneuropathy debut was 12 ± 8.3 days (range 1–30).

**Table 1:** Epidemiological data of GBS patients

	**Number of cases**	**(%)**
**Gender**		
**Male**	55	51.9
**Female**	51	48,1
**Age (years)**		
**<15**	15	14.2
**16-34**	24	22.6
**35-54**	28	26.4
**>55**	39	36.8
**Antecedent events**		
**Upper respiratory infection**	40	37.7
**Gastrointestinal infection**	29	27.4
**Vacunation**	2	1.9
**Others**	3	2.8
**None**	32	30.2
**Season**		
**Winter**	43	40.6
**Spring**	20	18.9
**Summer**	21	19.8
**Autumn**	22	20.8
**Motor deficit**		
**Mild (MRC 31–40)**	60	56.6
**Moderate (MRC 11–30)**	43	40.6
**Severe (MRC 0–10)**	3	2.8
**Cranial nerve involvement**	37	35.5
**Facial palsy uni/bilateral**	27	25.5
**MOE**	14	13.2
**Bulbar nerves**	4	3.8
**Ataxia**	8	7,5
**Other symptoms**		
**Sensory deficit**	31	29.2
**Pain**	33	31.1
**SNA**	9	8.5
**SIADH**	7	6.6
**Respiratory distress**	18	17
**GBS disability score**		
**Minor signs or symptoms**	12	11.3
**Walk without support**	41	38.7
**Walk with support**	16	15,1
**Bedridden or chair bound**	19	17.9
**Ventilated**	18	17
**Death attributed to SGB**	2	1.9

### Clinical data

A motor disorder at the admission was referred in 94 patients, with a variable degree. By classifying them according to the GBS disability score, 55% retained the ability to walk (grades 1, 2 and 3), unlike the remaining 45% which showed a severe affectation (grades 4, 5 and 6); Respiratory distress was present in 17% of patients. Pulmonary function was valued by the Peak Flow test in 50 patients, showed that values below 250 L/min were associated with a greater likelihood of requiring MV during the income (p < 0.05), independently of the presence of uni/bilateral facial palsy (p < 0.05). Time between symptom onset and admission was significantly lower in the severe cases (mean 5.17 days) compared with the mild ones (mean 8.87 days), so a faster progression could be postulated in the first ones (p = 0.053). Non-motor symptoms were described; the most frequent, neuropathic pain in 31% of patients, followed by sensory disturbances in 29%. Autonomic dysfunctions were found in 8.5% of cases. In 7% of the patients a syndrome of inappropriate antidiuretic hormone hypersecretion (SIADH) was diagnosed [26].

### Laboratory and neurophysiological findings

A lumbar puncture was made in 95 patients (90%) with an average delay of 15 ± 11.71 days (range 1–80) since the beginning of symptoms. Raised concentration of proteins was present in 80 patients (85%) with albuminocytological dissociation in 79 of them (85%).

Serologic studies were made in 101 patients (95%), in 8 (8%) cases CMV was the microorganism responsible for the GBS; in 5 (5%) *Mycoplasma Pneumoniae*, in 1(1%) EBV and 1 (1%) enterovirus; the remaining 85.1% of serology was negative.

NCS was made in 98 patients (92.5%) with a median period from the onset to the neurophysiological study of 20.73 ± 10.17 days (range 2–62). Resumed data of the neurophysiological test is shown in Table 2. In 57 (58%) a demyelinating pattern was found, 7 (7%) an axonal pattern, 3 (3%) were unexcitable, 2 (2%) were normal, and 29 (30%) didn’t fulfil diagnostic criteria for demyelinating lesion, but changes consistent with a peripheral neuropathy were present. Conduction blocks were present in 37 patients (37.8%). F- Responses were altered (absent or delayed) in 29 of 64 median (45%), in 16 of 35 ulnar (46%) in 4 of 8 tibial (50%) and in 34 of 40 (85%) peroneal nerves. H-reflex was affected in 26 of 48 (45%) cases evaluated. In sixth of the Miller Fisher syndromes a NCS was made, 1 of them (17%) was normal, and in the remaining 5 (83%) sensory conduction was affected. In 3 of 5 (60%) H reflex was absent. In the needle EMG examination signs of acute denervation were present in 44 of 86 (51%).

**Table 2:** Neurophysiological data of patients with GBS

	**N**	**CMAP (mV)**	**MDL (ms)**	**MCV (m/s)**	**n**	**SNAP (μV)**	**DL(ms)**	**SCV (m/s)**
**Median**	83	6,8 ± 5,6	6,4 ± 7,8	43,6 ± 17,9	85	7,03 ± 7,8	3,2 ± 3,4	43,6 ± 16,6
Min-Max		0,07-21,30	0,1 ± 61,0	0,10 ± 65,5		0,1-43,2	0,1-27,7	0,1-63,4
**Ulnar**	32	8,0 ± 7,8	3,4 ± 1,5	46,6 ± 17,4	72	3,9 ± 3,9	2,6 ± 1,9	43,2 ± 16,4
Min-Max		0,1-16,6	0,1-7,5	0,1-8,1		0,1-19,9	0,1-14,2	0,1-59,7
**Tibial post**	14	4,8 ± 5,9	6,7 ± 2,2	33,5 ± 15,6				
Min-Max		0,2-18,10	4,0-11,0	0,1-50,20				
**Peroneal**	87	3,6 ± 3,9	6,9 ± 5,1	35,9 ± 14,8				
Min-Max		0,1-15,7	0,1-31,4	0,1-54,1				
**Sural**					71	6,01 ± 5,4	2,6 ± 1,9	39,2 ± 15,5
Min-Max						0,1-27,4	0,1-18,0	0,1-57,2
**Sup Peroneal**					39	9,7 ± 8,2	2,2 ± 0,7	39,8 ± 12,7
Min-Max						0,1-37,4	0,1-3,8	0,13-8

The distribution of the different subtypes of GBS was: AIDP in 83%, acute motor and sensory axonal neuropathy (AMSAN) in 5.7%, acute motor axonal neuropathy (AMAN) in 1.9%, MFS in 8.5% and cranial multineuritis in 0.9%.

### Treatment, outcome and prognosis

Some kind of treatment was offered to 89 patients (84%): 88 received IVIg (83%) and 3 plasma exchange (2.8%); in 2 patients both treatments were dispensed sequentially. 16% of cases never started a treatment due to the mild symptoms or the long evolution of the disease.

At discharge, absent or minor deficits were observed in 38 patients (36%), 30 (28%) were able to walk for 10 meters without a help, 28 (26%) needed assistance to walk, 7 (7%) were bedridden and 1 (1%) needed respiratory support. Two patients died of the disease. Patients were followed in the outpatient clinic at 3, 6 and 12 months (Table 3).

**Table 3:** Proportion of patients, based on the GBS score, during the follow-up

	**Discharge**		**3th month revision**		**6th month revision**		**12th month revision**	
	**N**	**(%)**	**N**	**(%)**	**N**	**(%)**	**N**	**(%)**
**Healthy**	4	3,8	18	29,5	17	36,2	18	58,1
**Minor deficits or symptoms**	34	32,1	19	31,1	15	31,9	7	22,6
**Walk without support**	30	28,3	14	23	8	17	2	6,5
**Walk with support**	28	26,4	10	16,4	7	14,9	4	12,9
**Bedbridden or chairbound**	7	6,6	0		0		0	
**Ventilated**	1	0,9	0		0		0	
**Death**	2	1,9	0		0		0	

Some factors were analysed as possible predictors of a poor outcome: 1) patients with ages greater than 55 years were most affected at the admission (p = 0.027), with greater deficits at discharge (p = 0.2) and at the third (p = 0.1), sixth (p = 0.001) and twelfth month (p = 0.006); 2) severity at admission, score based on the GBS disability scale (disabling or non-disabling), was also associated with more disability at discharge (p < 0.001), and at the successive medical reviews at the third (p = 0.001) and the sixth month (p < 0.001); 3) cranial nerve involvement was related with greater deficits at discharge (p = 0.008) and 4) mechanical ventilation requirement showed greater sequels at discharge (p = 0.003), in the follow up the results were not statistically significant but a trend to associate with greater deficits was seen. Finally, there seems to be a trend towards a worse prognosis in those patients with axonal lesions in the conduction studies (p = 0.2) which is likely to be maintained throughout the evolution (Table 4).

**Table 4:** Possible predictor factors of a poor outcome

	**Discharge deficits**		**3th month revision deficits**		**6th month revision deficits**		**12th month revision deficits**	
	**Disabling**	**p**	**Disabling**	**p**	**Disabling**	**p**	**Disabling**	**p**
**Severity at admission**		**<0.001**		**0.001**		**<0.001**		**0.2**
**Non disabling**	11.3% (6/53)		20.6% (7/34)		7.7% (2/26)		11.8% (2/17)	
**Disabling**	60.3% (32/53)		63% (17/27)		61.9% (13/21)		28.6% (4/14)	
**Age**		**0.2**		**0.17**		**0.01**		**0.006**
**<55 years**	31.3% (21/67)		34.1% (15/44)		17.6% (6/34)		8% (2/25)	
**>55 years**	43.6% (17/39)		52.9% (9/17)		69.2% (9/13)		66.7% (4/69	
**MV**		**0.003**		**0.13**		**0.6**		**1**
**Yes**	66.7% (12/18)		66.7% (6/9)		42.9% (3/7)		25% (1/4)	
**No**	29.5% (26/88)		34.6% (18/52)		30% (12/40)		18.5% (5/27)	
**Axonal lesion at CNS**		**0.2**		**0.3**		**0.06**		**0.4**
**Demyelinating**	40.7% (22/54)		43.3% (13/30)		30.4% (7/23)		16,7% (3/18)	
**Axonal**	71.4% (5/7)		66,7% (4/6)		80% (4/5)		33.3% (1/3)	

Statistical analysis of the disabling deficits and the possible poor prognostic factors during the follow-up.

## Discussion

The present work has the limitations of a retrospective study based on hospital case-mix. The incidence is reported to be 0.6-2.4 cases per 100,000 per year [18,19,21]. Changes suffered in the last years in the attendance area of the hospital make incidence calculation complex and inaccurate; however, it appears to be of 1.68-2.46 per 100,000 per year.

There is no difference between gender [1,2]. The bimodal shape wasn’t present in our study [8-10], as there is a linear increase in the incidence with age [1,2,9,11,12,24]. GBS is considered a sporadic illness, without a seasonal cluster [1,9]; however, a trend to accrue in winter is shown in our series [11,12]. The infectious event is described to appear in 40-70% of patients [1-3,8-12]. In our series up to 70% of cases have had one, of which respiratory infection was the most frequent.

As in previous series, weakness and hypo/areflexia were the most frequent symptoms, followed by neuropathic pain and numbness. Hyponatremia, as a symptom of SIADH is not a classical manifestation of GBS; however, there are series in which are described to be present in up to 58% of the cases; in our review, it was found in 7% of our patients.

There isn’t a consensus about the neurophysiological values defining GBS and its variants [2,4,5,7,23,24,26-29]. It is accepted as demyelination parameters: motor conduction velocity (MCV) decrease, prolongation of motor distal latency, conduction blocks, temporal dispersion and increased F-wave latency [27]. It is reported that the first electromyographic changes are the alteration of F-wave and H-reflex response [2,22], altered both in our NCS reported as normal, probably due to the earliness of the exploration. In MFS, the CNS are normal in most cases; nonetheless, discrete changes in the sensory conduction or in H-reflex may be present [30-32], some authors postulate a damage in the afferent proprioceptive system as a pathophysiological basis [32].

As classically described, in our study illness prognosis is favourable; 81% of patients presented absent or minimum neurological deficits one year after the onset. Older age, illness severity in the acute phase, prior gastrointestinal infection and axonal injury in the CNS and mechanical ventilation requirement [15,20,33,34] are among the factors that have been advocated for poor prognosis. *Van Koningsveld* et al. defined a clinical prognostic scoring system for GBS outcome at 6 months, the “Erasmus GBS Outcome Score” or EGOS [15], it was based on the punctuation on the GBS disability score at 2 weeks from the admission, the history of diarrhoea and the age. Recently, *Walgaard et al.* have validated a modified EGOS (mEGOS) with the main difference being the use of the MRC sum score at admission and in the 7th day instead the GBS disability score [35], they claimed that the MRC sum score is more accurate, and the possibility of being used at admission could predict the future treatments. However, the mEGOS made on the 7th day after the admission show increased predictive value instead the one made on the first day [35]. However, although useful, the mEGOS passed on the first day of admission showed lower predictive ability than the one performed on the 7th day. In our study we demonstrate that, even in the first day of admission, lower scores on the GBS scale are associated with worse outcome and greater disability at discharge, 3 and 6 months. Respiratory distress is the leading cause of death in the acute phase, 20-30% requiring ventilatory support [20]. Many factors have been proposed as predictors of the future need for respiratory support, like forced vital capacity (FVC) < 60%, bulbar dysfunction, rapid progression of the illness, and difficulty raising the head [19,20]. *Van Doorn* et al. propose a regularly monitoring of the respiratory function initially every 2-4 h, and then every 6-12 h [1]. Although FVC is considered to be the gold standard test for detecting impaired ventilation it has some disadvantages, the requirement of portable spirometers in the acute phase due to the instability of the patient, the need for a minimum of preparation and knowledge of the technique by medical personnel and the higher cost. *Suárez et al*. describe a serie of 79 patients with neuromuscular diseases in which the Peak Flow test proved to be useful in the monitoring of expiratory muscle weakness [35]. In our hospital, patients were monitored by the Peak Flow test each 6 hours in the acute phase, being observed that values below 250 L/min predict the posterior need of respiratory support (p < 0.05), independently of the presence of facial palsy that could hinder the use of the test (p < 0.05), making the Peak Flow a safe, inexpensive, and widely-available test in the monitoring of patients with GBS.

## Conclusion

Our series is in concordance with those previously published. The seasonal cluster in winter is worth noting on which there is a great controversy. Regarding the outcome, our series reported a worse prognosis in patients with older age, severe deficits at the beginning, injured cranial nerves, requiring MV, and axonal lesion patterns in the NCS. Finally, project the Peak Flow-test as a useful predictive factor of respiratory failure by its availability, and easy management.

## Abbreviations

AIDP: Acute inflammatory demyelinating Polyradiculoneuropathy; AMAN: Acute motor axonal neuropathy; AMSAN: Acute motor and sensory axonal neuropathy; CB: Conduction blocks; CMAP: Conduction motor action potential; CMV: Cytomegalovirus; CSF: Cerebrospinal fluid; EBV: Ebstein-Barr virus; EMG: Electromyography studies; GBS: Guillain-Barré syndrome; HSV: Herpes-simplex virus; IVIg: Immunoglobulin; MRC: Medical research council; MFS: Miller-Fisher syndrome; MV: Mechanical ventilation; NCS: Nerve conduction studies; SIADH: Syndrome of inappropriate antidiuretic hormone hypersecretion; VZV: Varicella-Zoster virus.

## Competing interests

There are no competing interests in this manuscript.

## Authors’ contributions

IGS conceived of the study, participated in the design of the study and performed the statistical analysis, and coordination and drafted the manuscript. ISG participated in the design of the study. FJRR participated in the design of the study. JA conceived of the study, participated in the design of the study and coordination and drafted the manuscript. All authors read and approved the final manuscript.

## Pre-publication history

The pre-publication history for this paper can be accessed here:

http://www.biomedcentral.com/1471-2377/13/95/prepub
